# Supplementary material for: Specific Recognition of the 5′-Untranslated Region of West Nile Virus Genome by Human Innate Immune System
Source: Viruses. 2022 Jun 13;14(6):1282. doi: 10.3390/v14061282 (PMC9227302; doi:10.3390/v14061282)
Supplement: Supplementary file 1 [file viruses-14-01282-s001.zip › viruses-1734629-supplementary.pdf]

Supplementary Materials: Specific recognition of the 5′-untranslated region of West Nile Virus genome by human innate immune system

Emmanuelle Bignon [1,*], Marco Marazzi [2], Tom Miclot [1,3], Giampaolo Barone [3], and Antonio Monari [1,4,*]

Supplementary figures and tables

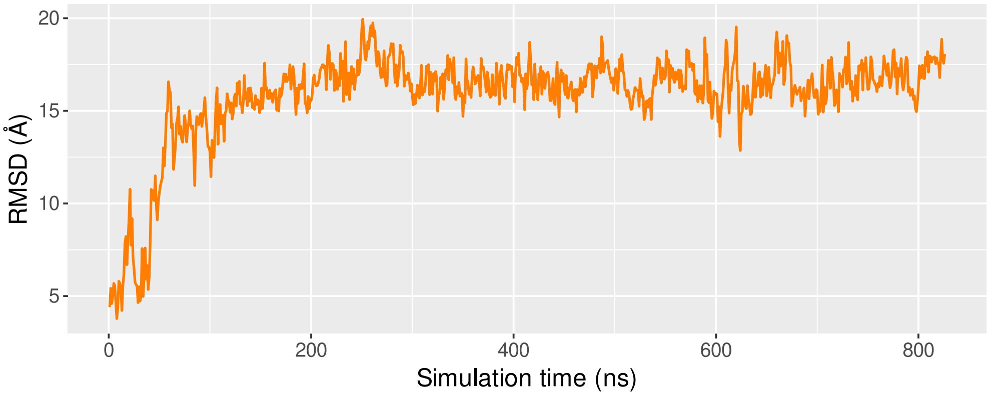

Figure S1. Evolution of the RNA root mean square deviation along the MD simulation of the isolated 5′ UTR system.

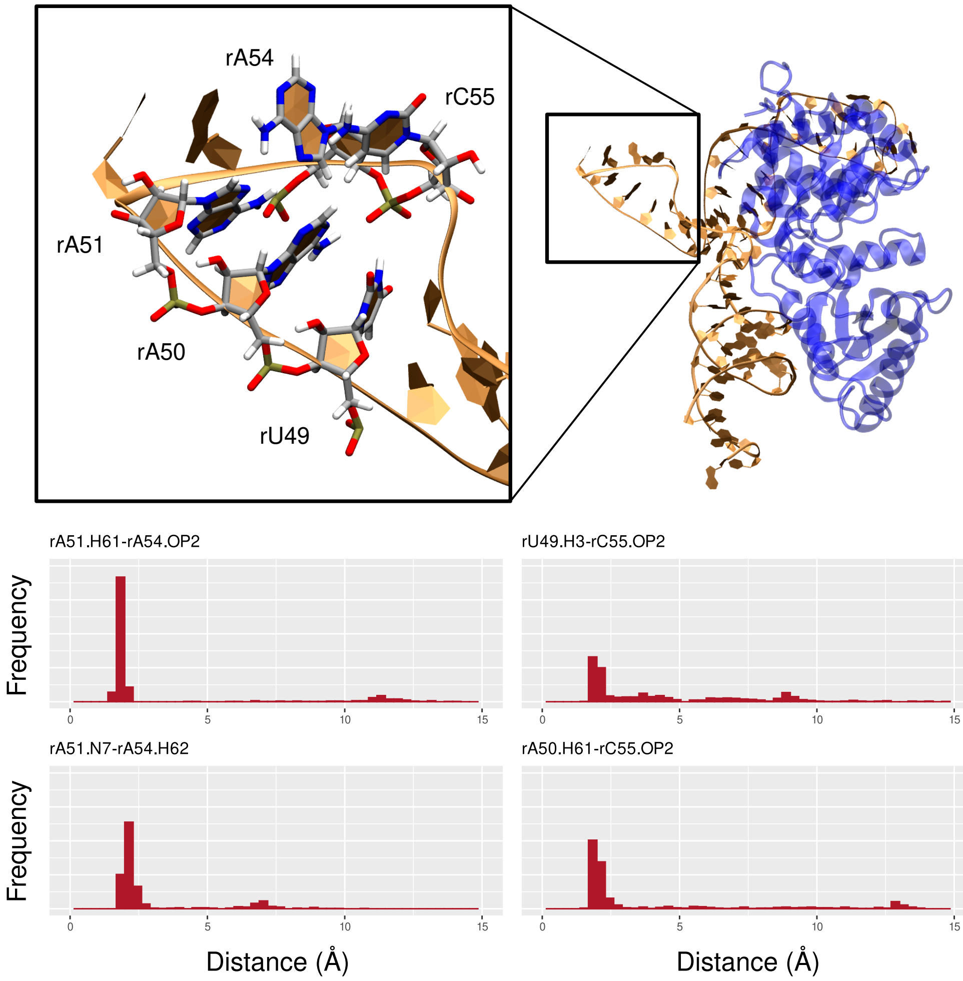

Figure S2. Representation of the nucleic acids involved in the major interactions within the lateral RNA loop in the OAS1+RNA complex system simulation (top), and frequencies of the associated distances (bottom).

Table S1. Percentage of occurrence of the five major clusters calculated from the isolated RNA and OAS1/RNA complex MD ensembles.

Cluster	RNA	RNA+OAS1
1	75 %	82 %
2	13 %	13 %
3	4 %	2 %
4	3 %	1 %
5	1 %	1 %
